# Disturbed Lipid Metabolism in Diabetic Patients with Manifest Coronary Artery Disease Is Associated with Enhanced Inflammation

**DOI:** 10.3390/ijerph182010892

**Published:** 2021-10-17

**Authors:** Katja Buschmann, Yves Gramlich, Ryan Chaban, Matthias Oelze, Ulrich Hink, Thomas Münzel, Hendrik Treede, Andreas Daiber, Georg Daniel Duerr

**Affiliations:** 1 Department of Cardiovascular Surgery, University Medical Center of the Johannes Gutenberg, University Mainz, Langenbeckstraße 1, 55131 Mainz, Germany; katja.buschmann@unimedizin-mainz.de (K.B.); Rayan.Chaban@unimedizin-mainz.de (R.C.); Hendrik.Treede@unimedizin-mainz.de (H.T.); 2 Department for Cardiology I, University Medical Center of the Johannes Gutenberg, University Mainz, Langenbeckstraße 1, 55131 Mainz, Germany; ygramlich@googlemail.com (Y.G.); matthias.oelze@unimedizin-mainz.de (M.O.); Ulrich.Hink@KlinikumFrankfurt.de (U.H.); tmuenzel@uni-mainz.de (T.M.); daiber@uni-mainz.de (A.D.)

**Keywords:** chronic disease, nutrition, diabetes mellitus, coronary artery disease, dyslipidemia, inflammation, oxidative stress

## Abstract

Background: Diabetic vasculopathy plays an important role in the pathophysiology of coronary artery disease (CAD) with oxidative stress as a strong mediator. This study aims to elucidate the underlying pathomechanisms of diabetic cardiac vasculopathy leading to coronary disease with an emphasis on the role of oxidative stress. Therefore, novel insights into antioxidant pathways might contribute to new strategies in the treatment and prevention of diabetic CAD. Methods: In 20 patients with insulin-dependent or non-insulin dependent diabetes mellitus (IDDM/NIDDM) and 39 non-diabetic (CTR) patients, myocardial markers of oxidative stress, vasoactive proteins, endothelial nitric oxide synthase (eNOS), activated phosphorylated eNOS (*p*-eNOS), and antioxidant enzymes, e.g., tetrahydrobiopterin generating dihydrofolate reductase (DHFR), heme oxygenase (HO-1), as well as serum markers of inflammation, e.g., E-selectin, interleukin-6 (IL-6), and lipid metabolism, e.g., high- and low-density lipoptrotein (HDL- and LDL-cholesterol) were determined in specimens of right atrial tissue and in blood samples from type 2 diabetic and non-diabetic patients undergoing coronary artery bypass graft (CABG) surgery. Results: IDDM/NIDDM increased markers of inflammation (e.g., E-selectin, *p* = 0.005 and IL-6, *p* = 0.051), decreased the phosphorylated myocardial *p*-eNOS (*p* = 0.032), upregulated the myocardial stress response protein HO-1 (*p* = 0.018), and enhanced the serum LDL-/HDL-cholesterol ratio (*p* = 0.019). However, the oxidative stress markers in the myocardium and the expression of vasoactive proteins (eNOS, DHFR) showed only marginal adverse changes in patients with IDDM/NIDDM. Conclusion: Dyslipidemia and myocardial inflammation seem to be the major determinants of diabetic CAD complications. Dysregulation in pro-oxidative enzymes might be attributable to the severity of CAD and oxidative stress levels in all included patients undergoing CABG.

## 1. Introduction

Diabetic cardiomyopathy is characterized by microstructural changes: myocardial oxidative stress leads to molecular and cellular inflammation, myofibroblast induction, as well as cardiomyocyte loss with subsequent replacement fibrosis [1,2,3]. Those changes are usually associated with diastolic dysfunction followed by symptomatic progressive systolic dysfunction and cardiac insufficiency [2].

Recently, animal studies have shed new light on this process, suggesting a role for an exaggerated oxidative cardiac damage and inflammation induced by diabetes mellitus in the progress of diabetic cardiomyopathy [2,4,5,6,7]. In addition, an increased inflammatory response was observed in diabetic patients [8,9] that was associated with vascular oxidative stress [10] and endothelial dysfunction [11,12]. In the long-term, this may play a major role in the formation of atherosclerotic lesions [13] and enhance the risk for cardiovascular disorders [14,15]. In addition, most cytokines are associated with higher cardiovascular risk and represent independent cardiovascular risk factors [16,17], which is a concept that was more recently explored for pharmacological targeting, e.g., within the “CANTOS trial” using “Canakinumab”, a monocloncal antibody against interleukin-1β in patients with previous myocardial infarction and high C-reactive protein (CRP) levels [18] and many other published or ongoing studies (reviewed in [19]). Of note, “Canakinumab” therapy also improved the cardiovascular prognosis of diabetic patients [20].

Measuring the level of oxidative stress in myocardium is a demanding process and requires the identification of suitable and reliable markers. Reduction in the cellular generation of tetrahydrobiopterin (BH_4_), a cofactor of the endothelial nitric oxide synthase (eNOS), is well known to be associated with oxidative stress, impaired endothelial function, and higher cardiovascular risk [21,22]_._ Furthermore, the heme oxygenase 1 (HO-1) is an important cardiac antioxidant protein, which is responsible for the production of biliverdin, bilirubin, and carbon monoxide [23,24,25]. These species have anti-atherosclerotic, anti-aggregatory, and vasodilatory properties, also acting as a potent antioxidant by the long-term induction of ferritin, which is responsible for iron storage and contributes to the prevention of Fenton-type oxidative damage. Its inducible isoform HO-1 is considered to be an important part of the cardiac antioxidant system, playing a role in stress response pathways [26,27]. Hyperlipidemia is a common phenomenon associated with type 2 diabetes mellitus caused by the insulin-dependency of lipoprotein lipase. Increased levels of low-density lipoprotein (LDL) lead to cholesterol deposition in arteries, while high levels of high-density lipoprotein (HDL) prevent cholesterol deposition [28,29].

Thus, the current study aims to validate the findings of animal studies that suggest that diabetes mellitus is associated with dyslipidemia, increased inflammation, and oxidative cardiac damage, e.g., diabetic cardiomyopathy, being strongly associated with the development of CAD [3,30]. The translational approach from animal studies to human myocardium represents a major requirement to understand the mechanisms of human disease, as our human atrial tissue samples are barely accessible and rare: In this work, we used human samples obtained from the right atrium from diabetic (IDDM/NIDDM) and non-diabetic (CTR) patients undergoing CABG due to CAD. We used cardiac markers to quantify the oxidative stress and antioxidant/vascular-regulatory proteins as well as serum markers of inflammation in the above-described patients with associated CAD.

## 2. Materials and Methods

### 2.1. Ethical Aspect

This study was conducted with permission of the ethical board of Rhineland-Palatinate, Germany (Register Nr. 837.104.08 (6100)) and after obtaining written consents of all participants. The work has been carried out in accordance with The Code of Ethics of the World Medical Association (Declaration of Helsinki). Samples of this study were already used for association studies of body mass index (BMI) with markers of oxidative stress and vascular function [31].

### 2.2. Patient Selection and Tissue Harvesting

The following inclusion criteria were defined: elective isolated CABG patients, sinus rhythmus; age under 85 years; absence of relevant valvular disease; absence of pulmonary hypertension; sufficient renal function; no known neoplasms or chronic systematic inflammatory diseases; and absence of severe comorbidity. The patients’ baseline characteristics are summarized in Table 1. For more details, see the published protocol where patients were grouped by different degree of adiposity and obesity (by BMI) and markers of oxidative stress as well as vascular function were associated with the BMI [31].

### 2.3. Blood Samples and Tissue Harvesting

Blood serum samples and myocardial tissues were obtained from 59 CABG patients as described [31]. Twenty patients had diabetes mellitus with oral medication or insulin-dependent type 2 diabetes mellitus (IDDM/NIDDM). The other 39 patients with regular blood glucose levels formed the control group (CTR). After thoracotomy, a full heparin dose was applied (400 IE/kg body weight) before cannulation of the aorta ascendens prior to the right atrium for cardiopulmonary bypass. Approximately 1 cm^3^ of the right atrial appendages were collected. Right atrial tissue is usually removed and discarded during cannulation of the right atrium in order to install cardiopulmonary bypass, as previously described [32]. Then, these tissue samples were immediately stored in ice-cold NaCl 0.9% solution and subjected to analysis.

### 2.4. Isolation of Cardiac Mitochondria and Mitochondrial Aldehyde Dehydrogenase 2 Activity

A detailed description of the mitochondria isolation method was previously published [31]. Briefly, the human cardiac tissues were homogenized in HEPES buffer and then centrifuged (1500× *g* for 10 min, followed by 2000× *g* for 5 min). Then, the supernatant was re-centrifuged (20,000× *g* for 20 min), and the resulting precipitate was resuspended in 1 mL Tris buffer. The amount of protein was quantified by the Lowry method. The activity of aldehyde dehydrogenase 2 (ALDH-2) in isolated heart mitochondria (final protein content 1 mg/mL) was determined by measuring the conversion of 6-methoxy-2-naphthylaldehyde (Monal 62) to the fluorescent naphthoic acid product by an HPLC-based assay as described [6,33].

### 2.5. Western Blot Analysis

Protein expression and modification was assessed by standard SDS-PAGE, Western blot analysis using established protocols [31,34]. Cardiac protein samples were analyzed by Western blot analysis for endothelial NO-synthase (eNOS, mouse monoclonal, 1:1000, BD Biosciences, Heidelberg, Germany), phospho-Ser1177-eNOS (rabbit polyclonal, 1:1000, Cell Signaling, Danvers, MA, USA), dihydrofolate reductase (DHFR, mouse monoclonal, 1 μg/mL, Abnova Corp., Heidelberg, Germany), monoclonal mouse heme oxygenase-1 (HO-1) (4 μg/mL, Stressgen, San Diego, CA, USA), and polyclonal rabbit β-actin (both 1:2500, Sigma-Aldrich, St. Louis, MO, USA) for normalization of loading and transfer. Detection and quantification were performed by enhanced chemiluminescence (ECL) with peroxidase conjugated anti-mouse/rabbit (GAM-POX/GAR-POX, 1:10,000, Vector Lab., Burlingame, CA, USA) secondary antibodies. Densitometric quantification of antibody-specific bands was performed with a ChemiLux Imager (CsX-1400M, Intas, Göttingen, Germany) and Gel-Pro Analyzer software (Media Cybernetics, Bethesda, MD, USA).

### 2.6. ELISA

Quantikine ELISA Immunoassays were performed according to the manufacturers’ instructions and as published [31,35]. The assays were obtained from R&D Systems using human serum samples: human interleukin-6 (IL-6) (Catalog Number D6050), human soluble vascular cell adhesion molecule-1 (sVCAM-1)/CD106 (CatalogNumber DVC00), human sE-Selectin/CD62E (DSLE00), and human CD40L/TNFSF5 (CatalogNumber DCDL40).

### 2.7. CRP, Blood Lipids, and Lipoproteins

Human C-reactive protein (CRP) was analyzed in the Department of Clinical Chemistry, University Hospital Mainz, Germany, using the daily routine facilities for in-patient care. Serum cholesterol, triglyceride, HDL, and LDL levels were analyzed in the Department of Clinical Chemistry, University Hospital Mainz, Germany, using the daily routine facilities for in-patient care. In addition, lipoproteins were also quantified by Field-Flow Fractionation (FFF), as described [6]. Lipoproteins in serum were also determined by HF5 (Superon GmbH, Dernbach, Germany). Briefly, Field-Flow Fractionation (FFF) is a well-known family of separation methods that vary in the physical nature of the force field applied to generate separation [36]. Asymmetric Flow Field-Flow Fractionation (AF4) is the most popular type of FFF. It employs a flat or cylindrical separation channel equipped with an ultrafiltration membrane and covers a wide separation range (1 nm to 1 μm). In HF5, the solvent is pumped through a porous fiber allowing a part of the flow to penetrate the wall, thus creating a cross flow that is perpendicular to the main solvent flow, which has a parabolic profile and is directed to the fiber outlet. The combination of the two forces applied eventually results in the separation of the sample compound according to their respective diffusion coefficient (i.e., their hydrodynamic radius or molar mass, respectively). Similar to AF4, HF5 has a wide range of applications. It allows the separation of molecules in solution and particles in the same separation run. The separation takes place without the use of a stationary phase as in column chromatography. Consequently, there is less danger of sample adsorption or physical plugging of the separation channel. Another advantage of this technique is the low sample dilution due to the small channel volumes (<100 μL) and low detector flow rates. The literature shows promising results for protein, nanoparticle, and even whole cell fractionation.

### 2.8. HPLC Assay for Dihydroethidium Oxidation Products

Superoxide was measured by a modified HPLC-based method to quantify ethidium and 2-hydroxyethidium levels, as previously described [37]. Briefly, myocardial mitochondria (0.2 mg/mL) were incubated with 50 μM dihydroethidium (DHE) for 30 min at 37 °C in PBS buffer and stored at −80 °C. Upon thawing, DHE oxidation products were extracted by the addition of 50% acetonitrile and 50% PBS, incubated (10 min), centrifuged (20 min at 20,000× *g*), and filtered (30 kDa Millipore Filter, 45 min at 16,000× *g*). A 50 μL sample of this supernatant was subjected to HPLC analysis and measured, based on a previously described method [38,39].

### 2.9. Statistical Analysis

Data are presented as mean ± SD. After evaluating the normal distribution of the data (using the Kolmogorov–Smirnov test), we applied an unpaired and two-tailed *t*-test or, where appropriate, a Mann–Whitney U-test for comparative analysis of reactive oxygen species (ROS) detection, protein activity, and expression and serum parameters (Prism for Windows version 8). *p* values < 0.05 were considered statistically significant.

## 3. Results

### 3.1. Myocardial Markers of Oxidative Stress

**Figure 1:**
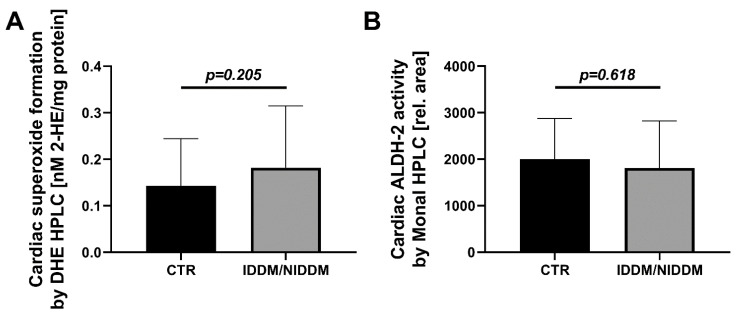
Markers of oxidative stress in myocardium. (**A**) Marginal higher concentration of mitochondrial superoxide formation (DHE HPLC) in the diabetic (IDDM/NIDDM) group versus control (CTR). (**B**) No difference in ALDH-2 activity after oxidative inactivation in the IDDM/NIDDM versus CTR group. 2-HE, 2-hydroxyethidium; ALDH-2, mitochondrial aldehyde dehydrogenase. Data are the mean ± SD of *n* = 38 (CTR) and 20 (IDDM/NIDDM) (**A**) and *n* = 16 (CTR) and 11 (IDDM/NIDDM) (**B**) patients. Mann–Whitney test in (**A**) and unpaired *t*-test in (**B**). CTR, control; IDDM, insulin-dependent diabetes mellitus type 2; NIDDM, non-insulin dependent diabetes mellitus type 2.

The primary markers of oxidative stress, direct measurement of mitochondrial superoxide formation and oxidative inactivation of ALDH-2, showed no significant changes of mitochondrial superoxide formation (DHE HPLC, *p* = 0.205) and cardiac ALDH-2 activity (Monal HPLC, *p* = 0.618) in the IDDM/NIDDM group (Figure 1).

### 3.2. Myocardial Vasoactive Regulatory Proteins and Antioxidant Enzymes

**Figure 2:**
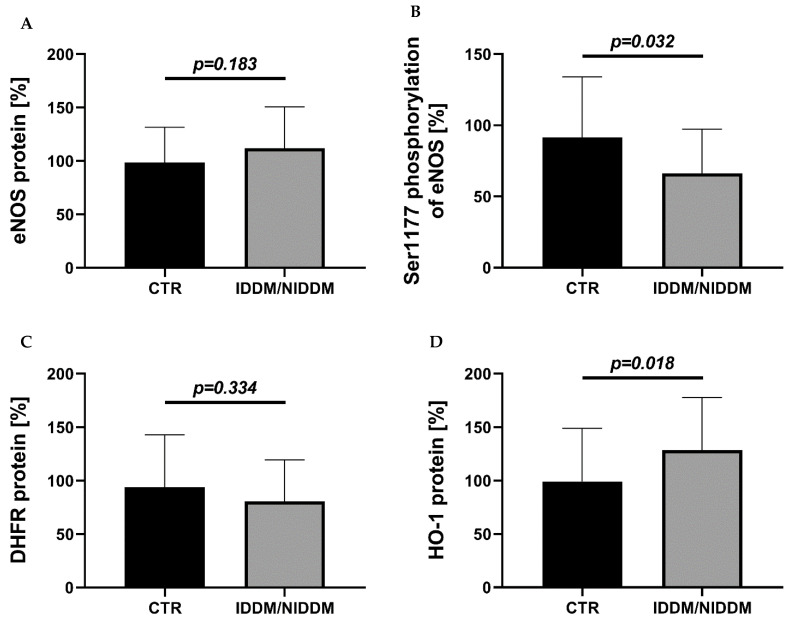
Expression of vasoactive regulatory protein and antioxidant enzyme/response in myocardium. (**A**) Absent upregulation of dysfunctional enzyme (eNOS protein) in the diabetic (IDDM/NIDDM) versus the control (CTR) group. (**B**) Significant decrease in activated eNOS (Ser1177 phosphorylation) in the IDDM/NIDDM versus CTR group. (**C**) Slightly lower DHFR expression in the IDDM/NIDDM versus CTR group. (**D**) Significant upregulation of the antioxidant stress-response enzyme HO-1 in the IDDM/NIDDM versus CTR group. Data are mean ± SD of *n* = 37–39 (CTR) and 17 (IDDM/NIDDM) (**A**,**B**) and *n* = 38 (CTR) and 16 (IDDM/NIDDM) (**C**) and *n* = 37 (CTR) and 16 (IDDM/NIDDM) (**D**) patients. Unpaired *t*-test in (**A**–**C**) and Mann–Whitney test in (**D**). CTR, control; IDDM, insulin-dependent diabetes mellitus type 2; NIDDM, non-insulin dependent diabetes mellitus type 2.

Cardiac eNOS protein showed a non-significant marginal increase in the diabetic subjects (*p* = 0.183) (Figure 2A). On the other hand, the phosphorylation of eNOS at serine 1177 (*p* = 0.032), an activation mark, was significantly decreased in the IDDM/NIDDM group (Figure 2B). Cardiac DHFR expression showed a non-significant marginal decrease in the diabetic subjects (*p* = 0.334) (Figure 2C). The antioxidant stress-response protein HO-1 was significantly upregulated in the IDDM/NIDDM group (*p* = 0.018) (Figure 2D).

### 3.3. Serum Markers of Inflammation

**Figure 3:**
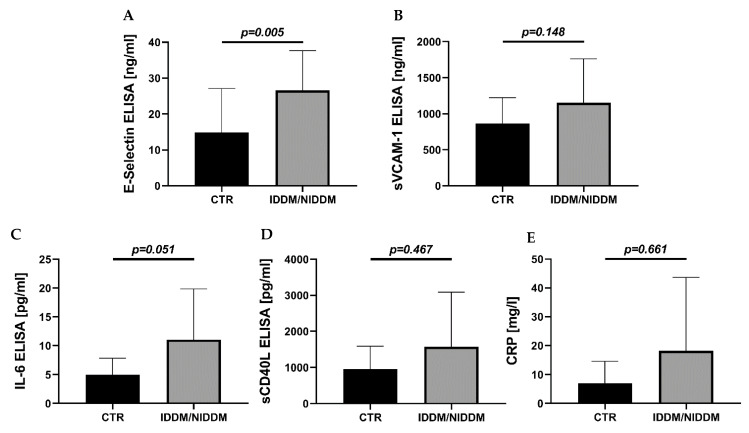
Serum markers of inflammation. (**A**) Significant raise of E-selectin in the diabetic (IDDM/NIDDM) versus the control (CTR) group. (**B**) Enhanced concentration of VCAM-1 in the IDDM/NIDDM versus CTR group (n.s). (**C**) Increased concentration of interleukin-6 (IL-6) in the IDM/NIDDM versus CTR group (n.s.). (**D**) Increase in concentration of CD40L and (**E**) C-reactive protein (CRP) in the DM versus CTR group (n.s.). Data are mean ± SD of *n* = 15 (CTR) and 11 (IDDM/NIDDM) patients (**A**–**D**) or *n* = 11–12 (**E**). Mann–Whitney test in (**A**,**D**,**E**) and unpaired *t* test in (**B**,**C**). CTR, control; IDDM, insulin-dependent diabetes mellitus type 2; NIDDM, non-insulin-dependent diabetes mellitus type 2.

While some inflammatory mediators were unchanged (sVCAM-1, *p* = 0.148 and sCD40L, *p* = 0.467 and CRP, *p* = 0.661), other serum markers of inflammation were increased in IDM/NIDDM (E-selectin, *p* = 0.005 and IL-6, *p* = 0.051) (Figure 3), pointing to a rather inflammatory and thrombotic phenotype in response to hyperglycemic conditions.

### 3.4. Serum Markers of Lipid Metabolism

**Figure 4:**
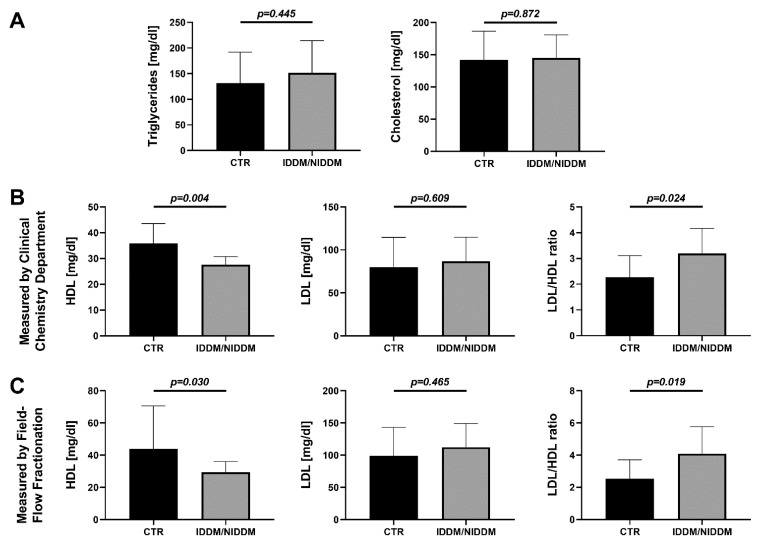
Serum markers of lipometabolism. (**A**) No obvious increase in triglycerides or total cholesterol in the IDDM/NIDDM versus CTR group. (**B**) (measurement by Clinical Chemistry Department) and (**C**) (measurement by Field-Flow Fractionation). Significant lower concentration of HDL-cholesterol together with a slightly higher concentration of LDL-cholesterol resulting in a significant raise of LDL/HDL ratio in the IDDM/NIDDM versus CTR group. Data are mean ± SD of *n* = 12 (CTR) and 11 (IDDM/NIDDM) (**A**,**B**) and *n* = 12 (CTR) and 10 (IDDM/NIDDM) (**C**) patients. Unpaired *t*-test in (**A**–**C**) [LDL and LDL/HDL ratio] and Mann–Whitney test in (**C**) [HDL]. CTR, control; IDDM, insulin-dependent diabetes mellitus type 2; NIDDM, non-insulin-dependent diabetes mellitus type 2.

Diabetic subjects showed no obvious increase in triglycerides (*p* = 0.445), total cholesterol (*p* = 0.872) (Figure 4A), or LDL-cholesterol (*p* = 0.609 in routine laboratory and *p* = 0.465 in Field-Flow Fractionation), but there was a significant lowered HDL-cholesterol (*p* = 0.03; Field-Flow Fractionation) and an imbalance between LDL and HDL, which was characterized by a higher LDL/HDL ratio (*p* = 0.024 and *p* = 0.019, respectively) in the IDM/NIDDM group (Figure 4B,C). The latter was mainly due to decreased HDL levels in the diabetic subjects, which was confirmed by two independent quantification methods for serum LDL and HDL (Clinical Chemistry routine measurement and Field-Flow Fractionation).

## 4. Discussion

The present study provides important insights into the contributing role of treated type 2 diabetes mellitus to the development of coronary artery disease in humans. Diabetic dyslipidemia is well known [40,41], and statin therapy should lower hyperlipidemia and prevent from the risk of CAD [40]. Here, we could show that dysregulated lipid metabolism and inflammation reactions are central pathomechanisms in diabetic vasculopathy. Our results provide evidence that the LDL-/HDL-cholesterol ratio and the E-selectin level in serum as a marker for vascular wall inflammation were raised in IDM/NIDDM patients compared to CTR with CAD. A higher LDL-/HDL-cholesterol ratio is a prognostic marker of higher atherosclerotic risk [42], and E-selectin is generally elevated in patients with CAD [43] and according to our present results further aggravated in patients with diabetes mellitus. As a general oxidative stress response in diabetes mellitus, the antioxidant HO-1 was upregulated, and there was less activated eNOS, resulting in endothelial dysfunction in accordance to animal experimental data reported previously [6].

In addition, we could show an increase in HO-1 protein levels in atrial tissue of diabetes mellitus. Gall et al. argued that hyperglycemia lowers HO-1 activity and increases superoxide production in the vasculature [44]. Interestingly, Oelze et al. have reported an upregulation of HO-1 in diabetic rats without therapy, assuming a general stress response, while rats under high-dose treatment with sodium-glucose cotransporter 2 inhibitor (SGLT2i) had a decline of HO-1 [6]. In this light, our data suggest either current antidiabetic therapy in diabetes mellitus protecting against HO-1 protein decrease or HO-1 providing a protective response, as plasma HO-1 levels are higher in patients with carotid plaques compared to healthy subjects, which probably indicates a possible protective response against carotid atherosclerosis, here against diabetic vasculopathy [44]. It remains unclear whether the HO-1 stress response is caused or affected by less activated eNOS in diabetes mellitus. The here-reported minor trend of eNOS protein upregulation in atrial tissue of diabetic patients is supported by similar observations in type 2 diabetic db/db mice [45,46]. Uncoupled eNOS leads to a lack of NO with disturbed endothelial function [47] and results in superoxide excess [48]. Probably HO-1 and eNOS protein upregulation compensates for the superoxide excess in IDDM/NIDDM, as we could not show an increased superoxide formation compared to CTR. However, we did clearly show less activated eNOS by decreased Ser1177 phosphorylation, as also shown in diabetic db/db mice [45,49] and an upregulation of HO-1 as an oxidative stress response. The predictive value of the regulatory enzymatic systems centered around eNOS was well documented in the past. Previously, it was shown that substituting BH_4_ improved the endothelial function in cell culture and animals [50] as well as patients with atherosclerosis [51]. Within the organelle, BH_4_ is produced by GTP cyclohydrolase I (GCH-1) and recycled from its oxidized form dihydrobiopterin (BH_2_) by DHFR [21,22]. Accordingly, BH_4_ levels or the expression levels of its main enzymatic sources, GCH-1 and DHFR, may be suitable to indirectly measure oxidative stress, although they are not changed significantly in the present study. Here, we observed a slight downregulation of DHFR protein in diabetic patients as also shown in diabetic db/db mice [52,53]. Altogether, our data support an uncoupled state of eNOS enzyme with futile compensatory upregulation of eNOS and DHFR proteins, which is compatible with the known impairment of endothelial function in diabetic subjects.

Dyslipidemia and cardiovascular inflammation are most likely the major pathomechanisms in diabetes mellitus with adverse impact on cardiovascular prognosis. Accordingly, it is not surprising that statin therapy improved the clinical outcome of patients with acute coronary syndromes, as this drug not only prevented dyslipidemia (decreased LDL levels) but also reduced inflammation (lower CRP levels), as shown by the PROVE IT-TIMI 22 study [54]. Statins have potent anti-inflammatory and antioxidant pleiotropic effects (e.g., via induction of NRF2/HO-1 pathway [55,56]), also in patients with diabetes [57,58,59,60]. Statins therapy also suppresses the activation of NADPH oxidase [61] and uncoupling of eNOS as well as subsequent endothelial dysfunction in animal models [62], all of which contribute to the beneficial antioxidant profile of statins [63]. In addition, statins mobilize endothelial progenitor cells, which could contribute to efficient repair of the endothelium, better recovery of coronary vessels from atherosclerotic insults, and thereby improved prognosis of patients with coronary artery disease [64].

## 5. Limitations of the Study

In the present study, we are talking about well-treated diabetes mellitus. We suggest the presence of a successful treatment without hyperglycemia in the IDDM/NIDDM group, although the proof by HbA1c < 6.5% was missed. Nevertheless, we can proclaim that patients with CAD and IDDM/NIDDM undergoing CABG should regularly present to a cardiac specialist, and medication for cardiovascular risk factors should be effective. The availability of left ventricular (LV) myocardium is rare and ethical considerations prohibit its use, as LV biopsies can be associated with severe bleeding and scar formation in the “working myocardium” and are thus considered harmful for patients. For this reason, our choice of myocardial specimen from the right atrial appendages has been reported before [32]. Due to the multiple measurements requiring large volumes, plasma samples were pooled for the ELISA assays in Figure 3 and for the determination of lipid parameters in Figure 4, explaining the reduced number of independent measurements for these parameters. The same applies for ALDH-2 activity measurement that also required the pooling of some of the heart samples. Finally, for protein expression, one to four data points are missing in each group due to insufficient blot quality (e.g., air bubbles caused incomplete transfer of the protein bands from the gel to the membrane during the Western blot procedure or a gel pocket was left empty before SDS-PAGE).

## 6. Conclusions

Inflammation and lipoprotein dysregulation remain the main triggers of diabetic coronary occlusive disease despite antidiabetic pharmacotherapy. Adjuvant pharmacological substances such as statins acting as anti-inflammatory agents and lipid blockers might diminish the detrimental effect of diabetes mellitus on the coronary inflammation and atherosclerosis. However, further studies are required to evaluate their true therapeutic value.

## Figures and Tables

**Table 1:** Patients’ baseline characteristics. Summary of preoperative patients’ characteristics. Values are expressed as mean ± SD or as percentage (in brackets). Significant changes are displayed in italics. Abbreviations: ACE, angiotensin-converting enzyme; AT1, angiotensin-1; BMI, body mass index. Chi^2^ test was performed for categorial variables, and an unpaired, two-tailed *t*-test was applied for comparison of metric variables. Significant changes are displayed in italics.

	CTR	IDDM/NIDDM	*p*-Value
Age (years)	65.15 ± 9.08	66.90 ± 9.83	0.5782
Female (%)	61.54%	61.11%	0.9754
Height (cm)	1.694 ± 0.081	1.681 ± 0.104	0.5856
Body weight (kg)	80.62 ± 13.5	79.56 ± 12.6	0.7794
BMI (ratio)	27.99 ± 3.62	28.43 ± 5.60	0.7234
Waist circumference (cm)	102.4 ± 8.69	102.6 ± 9.53	0.9257
Hip circumference (cm)	100.5 ± 6.49	104.9 ± 8.59	0.1357
Waist/Hip ratio	1.018 ± 0.077	0.986 ± 0.096	0.3452
Oral anti-diabetics (%)	0.00%	88.89%	*<0.0001*
Insulin dependence (%)	0.00%	16.67%	*0.0088*
ACE inhibitors (%)	35.90%	50.00%	0.3131
AT1 antagonists (%)	12.82%	22.22%	0.3656
Beta-blocker (%)	58.97%	55.56%	0.8080
Calcium-channel blocker/%)	20.51%	16.67%	0.7323
Spironolactone (%)	2.56%	0.00%	0.4931
Statins (%)	64.10%	50.00%	0.3131

## Data Availability

The data used to support the findings of this study are available from the corresponding author upon request.
